# Analysis of Documentation Speed Using Web-Based Medical Speech Recognition Technology: Randomized Controlled Trial

**DOI:** 10.2196/jmir.5072

**Published:** 2015-11-03

**Authors:** Markus Vogel, Wolfgang Kaisers, Ralf Wassmuth, Ertan Mayatepek

**Affiliations:** ^1^ University Children’s Hospital Düsseldorf Department of General Pediatrics, Neonatology and Pediatric Cardiology Heinrich-Heine-University Düsseldorf Germany; ^2^ Center of Bioinformatics and Biostatistics Mathematical Institute Heinrich-Heine-University Düsseldorf Germany; ^3^ University Hospital Düsseldorf Staff Unit Quality Management and Patient Safety Heinrich-Heine-University Düsseldorf Germany

**Keywords:** electronic health record, automatic speech recognition, randomized controlled trial

## Abstract

**Background:**

Clinical documentation has undergone a change due to the usage of electronic health records. The core element is to capture clinical findings and document therapy electronically. Health care personnel spend a significant portion of their time on the computer. Alternatives to self-typing, such as speech recognition, are currently believed to increase documentation efficiency and quality, as well as satisfaction of health professionals while accomplishing clinical documentation, but few studies in this area have been published to date.

**Objective:**

This study describes the effects of using a Web-based medical speech recognition system for clinical documentation in a university hospital on (1) documentation speed, (2) document length, and (3) physician satisfaction.

**Methods:**

Reports of 28 physicians were randomized to be created with (intervention) or without (control) the assistance of a Web-based system of medical automatic speech recognition (ASR) in the German language. The documentation was entered into a browser’s text area and the time to complete the documentation including all necessary corrections, correction effort, number of characters, and mood of participant were stored in a database. The underlying time comprised text entering, text correction, and finalization of the documentation event. Participants self-assessed their moods on a scale of 1-3 (1=good, 2=moderate, 3=bad). Statistical analysis was done using permutation tests.

**Results:**

The number of clinical reports eligible for further analysis stood at 1455. Out of 1455 reports, 718 (49.35%) were assisted by ASR and 737 (50.65%) were not assisted by ASR. Average documentation speed without ASR was 173 (SD 101) characters per minute, while it was 217 (SD 120) characters per minute using ASR. The overall increase in documentation speed through Web-based ASR assistance was 26% (*P*=.04). Participants documented an average of 356 (SD 388) characters per report when not assisted by ASR and 649 (SD 561) characters per report when assisted by ASR. Participants' average mood rating was 1.3 (SD 0.6) using ASR assistance compared to 1.6 (SD 0.7) without ASR assistance (*P*<.001).

**Conclusions:**

We conclude that medical documentation with the assistance of Web-based speech recognition leads to an increase in documentation speed, document length, and participant mood when compared to self-typing. Speech recognition is a meaningful and effective tool for the clinical documentation process.

## Introduction

The diagnostic and therapeutic procedures of medical professionals lead to a vast number of observations and decisions, which must be documented correctly to ensure the documentation of the medical course, fulfillment of legal aspects, quality reporting, and billing. The electronic health record (EHR) system plays a critical role in documenting the clinical treatment procedure. The electronic availability of clinical data improves readability, administration, safety, and communication during the course of treatment. On the other hand, electronic health records can interrupt clinical workflows and the treatment procedure, conceivably because of limited availability at bedside. These aspects lead to different beliefs and experiences of health care professionals concerning the general quality of electronic health record systems, clinical day-to-day usability, and user satisfaction [1].

Automatic speech recognition (ASR) systems are believed to facilitate documentation while using the EHR. In clinical specialties with high demands for structured documentation (eg, radiology, pathology), ASR systems are a standard tool today although several studies on ASR in the field of radiology reflect a large amount of heterogeneity [2]. In general, when using a current front-end ASR system, the dictated text immediately appears in visible characters on the screen, and medical documentation can be finalized as soon as it has been entered. As a result, the report is available without delays due to corrections or transport of the report.

Without the availability of a front-end ASR system, the user is forced to wait for a transcriptionist to enter content, to wait for a back-end ASR system to finish the job, or to enter it manually through a keyboard, mouse, or touch screen including all necessary corrections. This already leads to an avoidance of individual documentation by inserting copied text blocks (ie, copy and paste). In an analysis of clinical documentation of an intensive care unit, 82% of documentation contained at least 20% inserted text blocks [3]. Clinical information can be lost due to insufficient adaptation and weighting of the inserted content. Besides, unforeseeable legal issues could be suspected [4].

Front-end ASR systems require the user to interact directly. Since ASR systems compare the user’s audio information with predefined patterns, recognition accuracy depends on correct grammar, consistent pronunciation, and constant feedback on new words or abbreviations. Therefore, users are urged to correct errors during report generation interactively in order to feed machine learning mechanisms.

Comparative analysis and synthesis of studies covering the usefulness of ASR in various clinical settings is challenging due to a narrative presentation of the results [5]. In 2003, a randomized controlled trial was undertaken outside radiology to compare back-end speech recognition with standard transcription, which failed to find an overall benefit [6]. Now, with the advent of new technologies, front-end ASR is available instantly in all areas and specialties of medicine in different languages [7]. But the effects of using current front-end ASR in a clinical setting on documentation speed, document length, and physician satisfaction are not known, thereby indicating the need for explorative studies on the topic.

We hypothesize that the addition of a Web-based, front-end ASR system to the clinical documentation process leads to an increase in documentation speed and documentation amount, and thereby increased physician satisfaction. To measure the effects of using a Web-based, front-end ASR system on documentation speed, document length, and physician satisfaction, we conducted a prospective randomized controlled trial. Documentation time, the number of documented characters, and physician satisfaction have been analyzed for keyboard and speech input in the German language. No changes have been made to any other aspect of the clinical documentation process.

## Methods

The study was not registered in a World Health Organization (WHO)-accredited trial registry since there was no applicable biomedical or health outcome conforming to any human subject or ethics review regulations, or regulations of the national or regional health authority.

### Study Design

Physicians from the Department of Pediatrics and the Department of Trauma Surgery, Düsseldorf University Hospital, Germany, were asked to participate in morning meetings. Two participants were asked to participate via personal communication. Enrollment was possible over a period of 30 days. The inclusion criteria were clinical activity of the participating physicians and documentation of at least two clinical reports within the study period.

All participants signed their informed consent forms. Each participant was known to the study team in person. Through the enrollment, the physician chose an individual username and password, not known to the study team, to access the study website. Thereafter, the users identified themselves using a username and a password. After written informed consent was obtained and the privacy policy signed, the password-protected, browser-based, study analysis home page was activated. The username was replaced with a number when storing study information in the database. The study was conducted by approval from, and according to requirements of, the Health Privacy Commissioner of Düsseldorf University Hospital (see Multimedia Appendix 1 for the CONSORT-EHEALTH checklist).

During a short, standardized technical training session, all participants documented a uniform standard text once using speech and once using a keyboard to assess individual speed levels. After that, no further contact between the study team and the participants occurred until the end of the study period of 120 days.

All participants were asked to do everyday clinical documentation in a browser’s text area. To complete a study step, the participant opened a webpage, logged in, and documented the clinical finding, report, or discharge letter in the browser’s text area. After completion, the text was manually copied into the EHR. For each study step, the length of the text, documentation time including necessary corrections, correction-associated usage of keyboard, and physician’s moods were captured using JavaScript. Each participant received the intervention in a random sequence. For each study step, log-in, or refresh of the webpage, a randomization occurred between the availability of speech recognition and the keyboard, or the keyboard alone.

**Figure 1:**
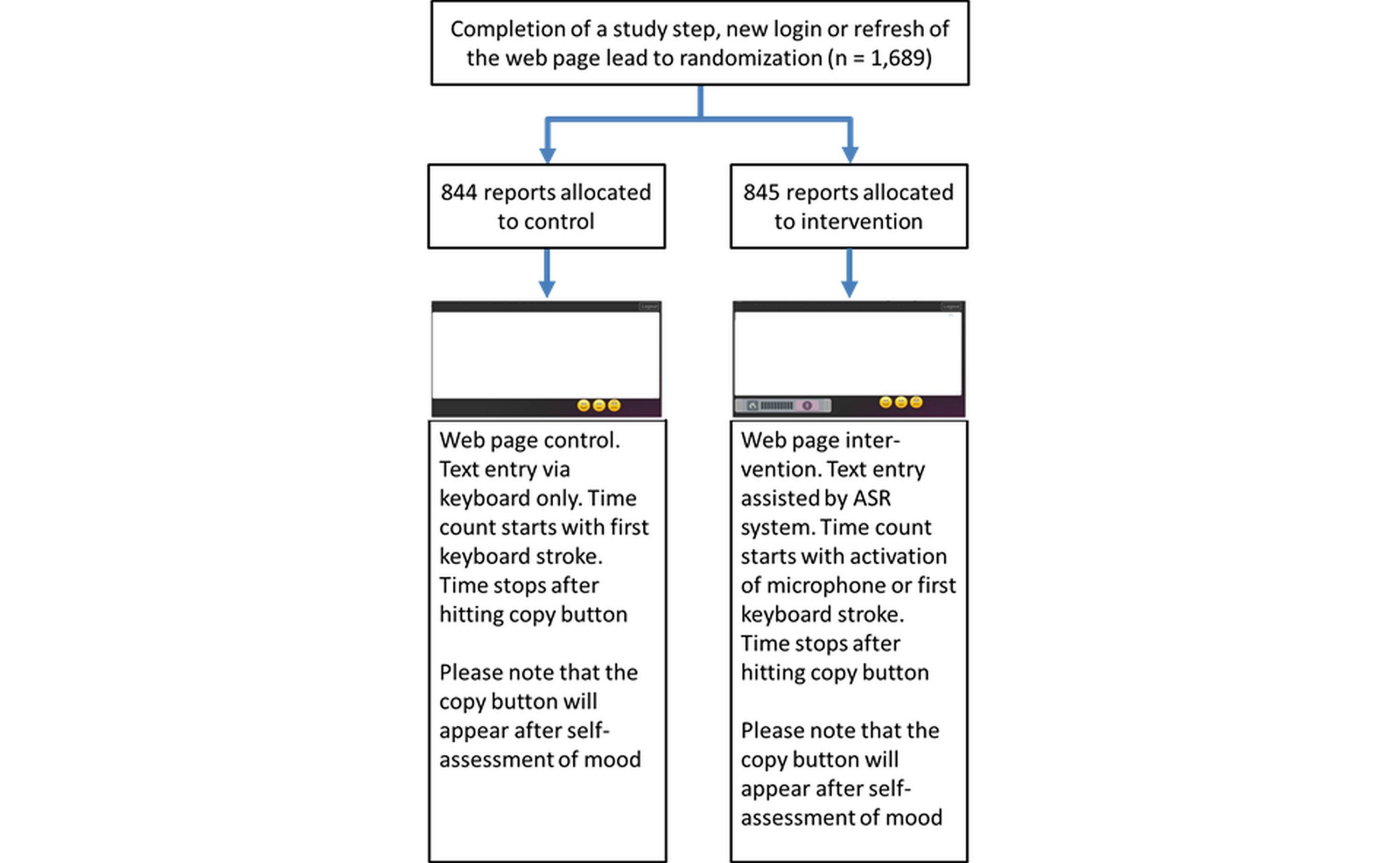
Completion of a study step (ie, clinical report), webpage layout of intervention and control, randomization procedure, and time measurement. Please note the existence of a speech plug-in during the intervention (grey bar, lower left corner of intervention webpage). Starting time count also starts the other counters used (ie, delete key, backspace key, arrow keys, mouse left click). The copy button will appear after the participant hits a smiley for self-assessment of mood. This action copies the text onto the clipboard for further usage in the EHR.

After all necessary corrections (misspellings, misrecognitions, etc) to achieve a correct text, the study step (ie, the clinical report) was finished by hitting one of three smileys to indicate the mood. The physician's mood was measured using a 3-point scale (1 = good, 2 = moderate, 3 = bad) by online self-assessment on the study webpage. Hitting one of three smileys lead to the appearance of a copy button. Finalization of a study step and the transfer of captured data to the storage database were achieved by hitting the copy button. This action placed the text in the clipboard and simultaneously triggered a new randomization. Depending on the randomization result, the browser loaded a script that enabled medical speech recognition in addition to conventional keyboard text entry. Closing the session without hitting the copy button or direct log-out lead to exclusion of the report for further analysis (see Figure 1).

Writing speed was calculated by the number of characters per minute. The underlying time frame was the text entry time and corrections until finalization of the document. Numerical measures are mentioned in the text as mean (SD). To reduce the impact of technical artifacts (eg, by inserting text blocks or by interrupting text entry without finalizing the study documentation step), documentations with greater than 1000 characters per minute, more than 1-hour documentation time, or fewer than 10 characters have been excluded.

During the study period, a Web-based medical speech recognition system has been used (Nuance SpeechAnywhereServices Browser SDK, SpeechAnywhereServices 1.6/ SpeechMagic Version 7 Release 4 FP4, MultiMed 510.706). The system was available on any clinical desktop computer having a microphone, Microsoft Silverlight installed, and access to the network. No modifications to the physician’s computer were made except the addition of a USB microphone (Samson Go Mic clip-on USB microphone). The only limitation was the restriction of usage for medical documentation only. The participants conducted their documentation based on their own needs. They were not allowed to use the system for private communication. It was not possible to insert text blocks by voice commands.

For each report, information including a time stamp had been saved for further analysis. The study information contained the following: current number, user ID, time stamp, session time, delete key count, backspace key count, arrow key count, mouse left-click count, total number of characters, self-assessment of mood, and type of session (intervention or control). The information was transmitted to a database using a Secure Sockets Layer (SSL) protocol. The end point of the study was the end of the study period.

### Statistical Analysis

Calculation of numerical results, statistical tests, and creation of images were performed using R version 3.2.1 (The R Foundation, Vienna, Austria) [8] using a permutation test.

## Results

During the recruitment period, 40 physicians asked for participation. Out of 40 physicians, 37 (93%) met the inclusion criteria. Out of 37 participants, 7 (19%) could not participate after initial enrollment because of organizational reasons. Out of 30 participants, 2 (7%) were excluded later because fewer than two documents were completed using the study system.

**Figure 2:**
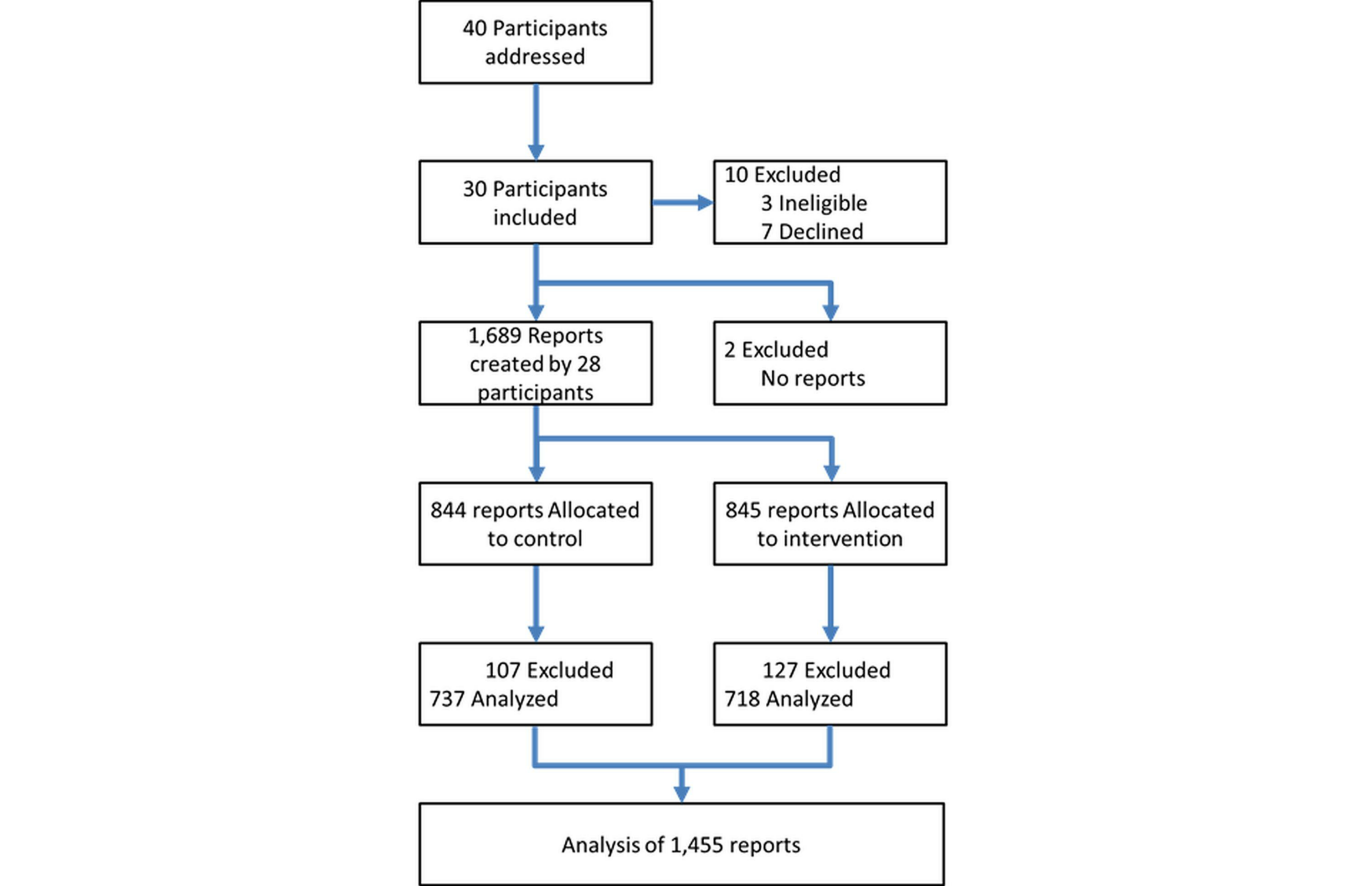
CONSORT-EHEALTH flowchart of enrollment, participants, and report status.

The 28 (100%) final participants were comprised of 21 (75%) interns and 7 (25%) senior physicians. A total of 17 out of 28 (61%) participants were male and 11 (39%) were female. A total of 22 out of 28 (79%) participants were from a nonsurgery department and 6 (21%) were from a surgery department (see Table 1). All participants were native German speakers. No participant was a trained typist or had professional exposure to speech recognition systems before (see Figure 2).

**Table 1:** Participant characteristics (n=28).

Participant characteristics	n (%)
All participants	28 (100)
Male	17 (61)
Female	11 (39)
Number of senior physicians	7 (25)
Surgery	6 (21)
Nonsurgery	22 (79)

**Figure 3:**
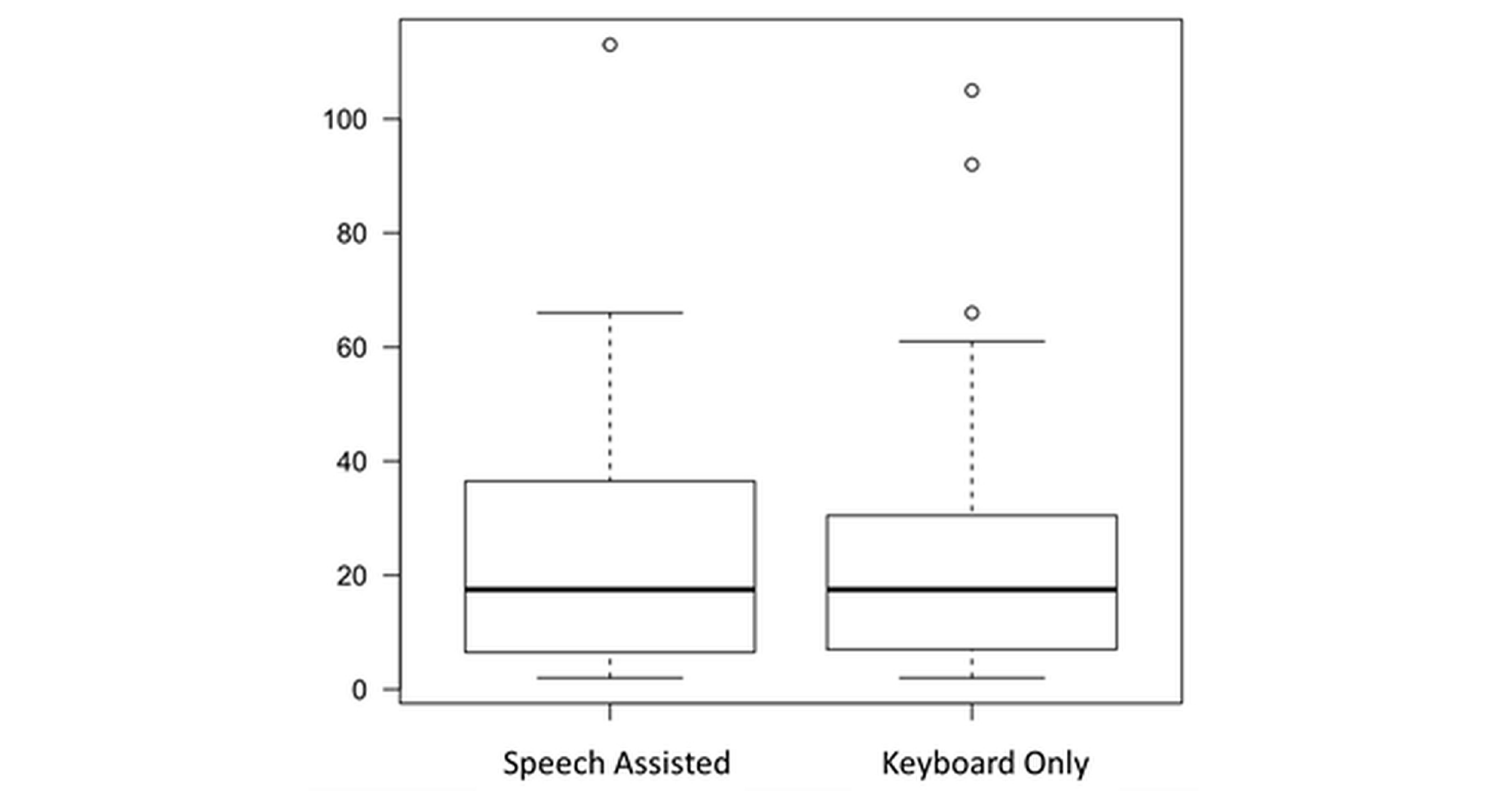
Box plot of the number of documentations per participant (total n=1455).

Over a period of 120 days, 1455 of 1689 (86.15%) recorded clinical reports from 28 participants met the inclusion criteria. A total of 234 reports out of 1455 (16.08%) were excluded because documentation speed was greater than 1000 characters per minute, documentation time was more than 1 hour, or the report contained fewer than 10 characters (see Figure 2). A total of 718 out of 1455 (49.35%) clinical reports were done using speech and 737 (50.65%) were done using the keyboard alone. Figure 3 shows the number of documentations per participant.

**Figure 4:**
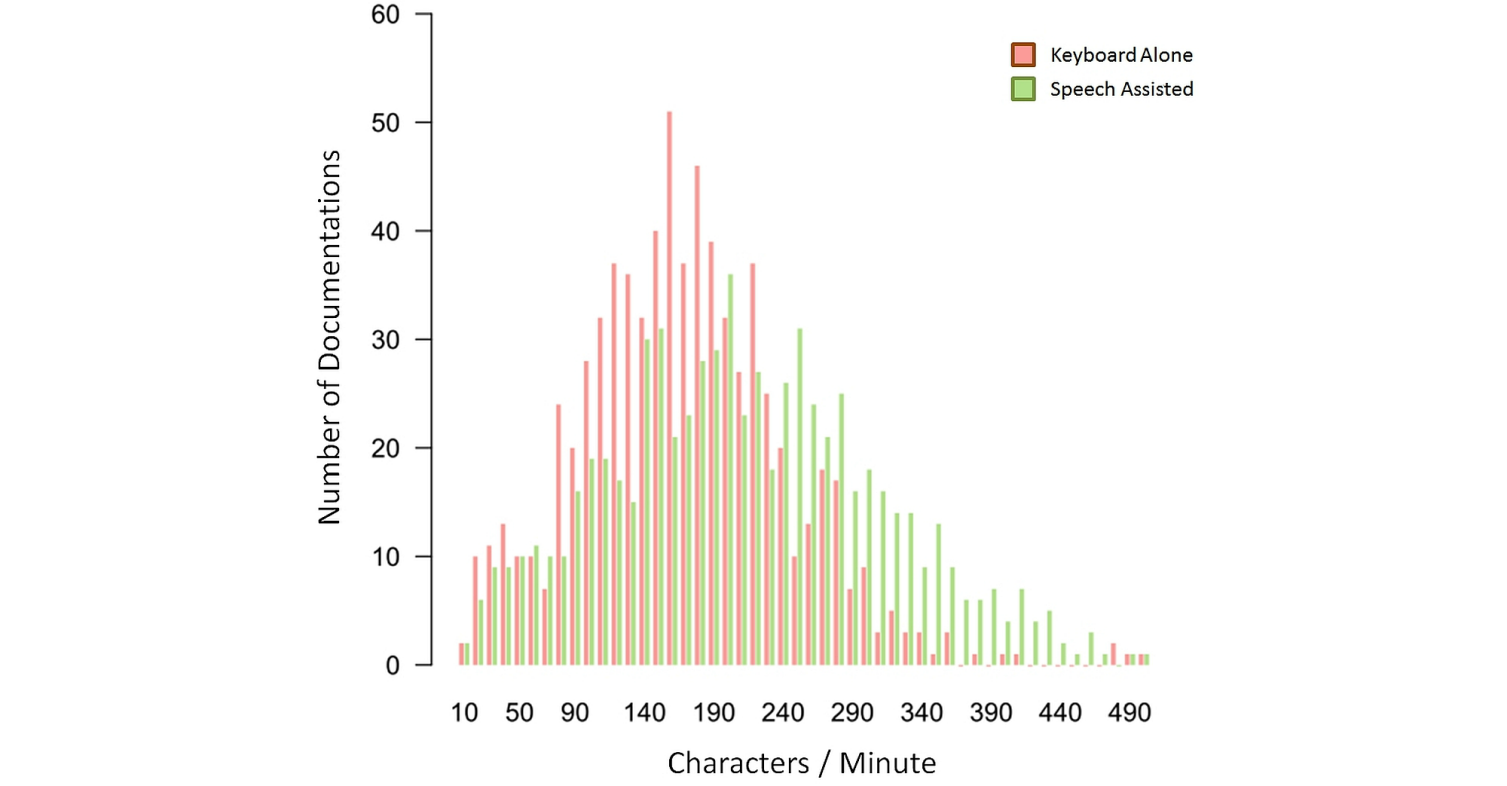
Distribution of documentation speed in characters per minute.

The average documentation speeds until the finalization of the report, including all corrections, were 173 (SD 101) characters per minute in the keyboard only (control) group, and 217 (SD 120) characters per minute in the speech-assisted (intervention) group. The documentation speed was increased by 25.7% in the speech group (*P*=.04, permutation test). The distribution of speed values is shown in Figure 4. Using the keyboard exclusively, an average of 356 (SD 388) characters per report were entered compared to 649 (SD 561) characters using speech entry. After the documentation, the physicians' average mood ratings were 1.6 (SD 0.7) using keyboard alone and 1.3 (SD 0.6) when using speech recognition (*P*<.001, permutation test). Table 2 shows a complete reference of captured data for productive use during the study period, and Table 3 shows a complete reference of captured data of standardized text.

**Table 2:** Captured data during productive use (n=1455)^a^.

Captured data: productive use	Keyboard only	Speech assisted
Number of reports/documentations, n (%)	737 (50.65)	718 (49.35)
Total number of characters	262,080	465,785
Total documentation time	37h 18min	55h 24min
Number of characters per report, mean (SD)	356 (388)	649 (561)
Number of delete key strokes^b^, mean (SD)	0.3 (1.2)	4.2 (9.5)
Number of backspace key strokes^b^, mean (SD)	25.7 (41.8)	10.3 (16.5)
Number of arrow key strokes^b^, mean (SD)	3.0 (7.1)	5.8 (15.2)
Number of mouse left clicks^b^, mean (SD)	2.8 (4.0)	11.4 (13.6)
Mood rating (1=good, 2=moderate, 3=bad), mean (SD)	1.6 (0.7)	1.3 (0.6)

^a^Please note the absolute numbers in Table 2 versus the relative numbers in Figure 6.

^b^The listed key strokes are necessary correction events to produce a final report.

**Table 3:** Captured data during standard text entry (n=60)^a^.

Captured data: standard text^b^	Keyboard only (n=30), mean (SD)	Speech assisted (n=30), mean (SD)
Duration (s)	376 (176)	339 (175)
Number of characters	939 (10)	956 (8)
Number of delete key strokes	0.8 (2.3)	5.6 (8.0)
Number of backspace key strokes	26.8 (15.0)	14.8 (15.7)
Number of arrow key strokes	6.2 (10.6)	13.9 (22.5)
Number of mouse left clicks	3.8 (4.4)	11.3 (10.1)
Mood rating (1=good, 2=moderate, 3=bad)	1.6 (0.7)	1.3 (0.6)

^a^Please note the absolute numbers in Table 3 versus the relative numbers in Figure 6.

^b^Each participant entered the standard text twice (control method and intervention method) and applied corrections to generate a correct text.

**Figure 5:**
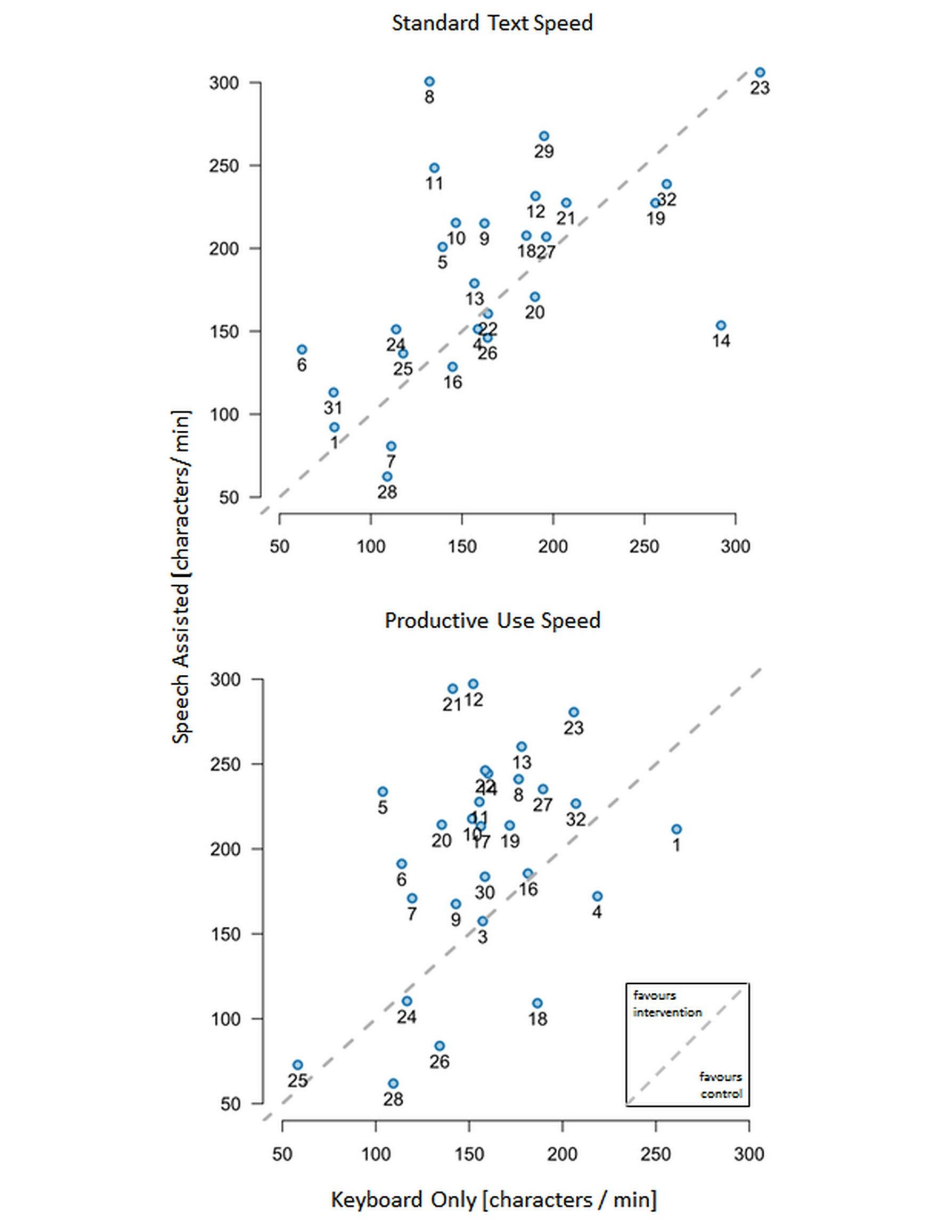
Per person analysis of documentation speed while documenting a standard text and during productive use in characters per minute. Each dot represents one participant. The location of the dot indicates the documentation speed of control and intervention. A location above the dotted line indicates a gain in speed using the intervention (speech-assisted documentation). The dots representing the standard text consist of one initial documentation pair while the dots representing productive use consist of all available data for each participant. The labeling of dots with numbers is for better comparison of both plots within the figure. Please note that documentation of the standard text reflects the individual’s typing capabilities on the x-axis and the individual’s initial capabilities in using the ASR system on the y-axis.

**Figure 6:**
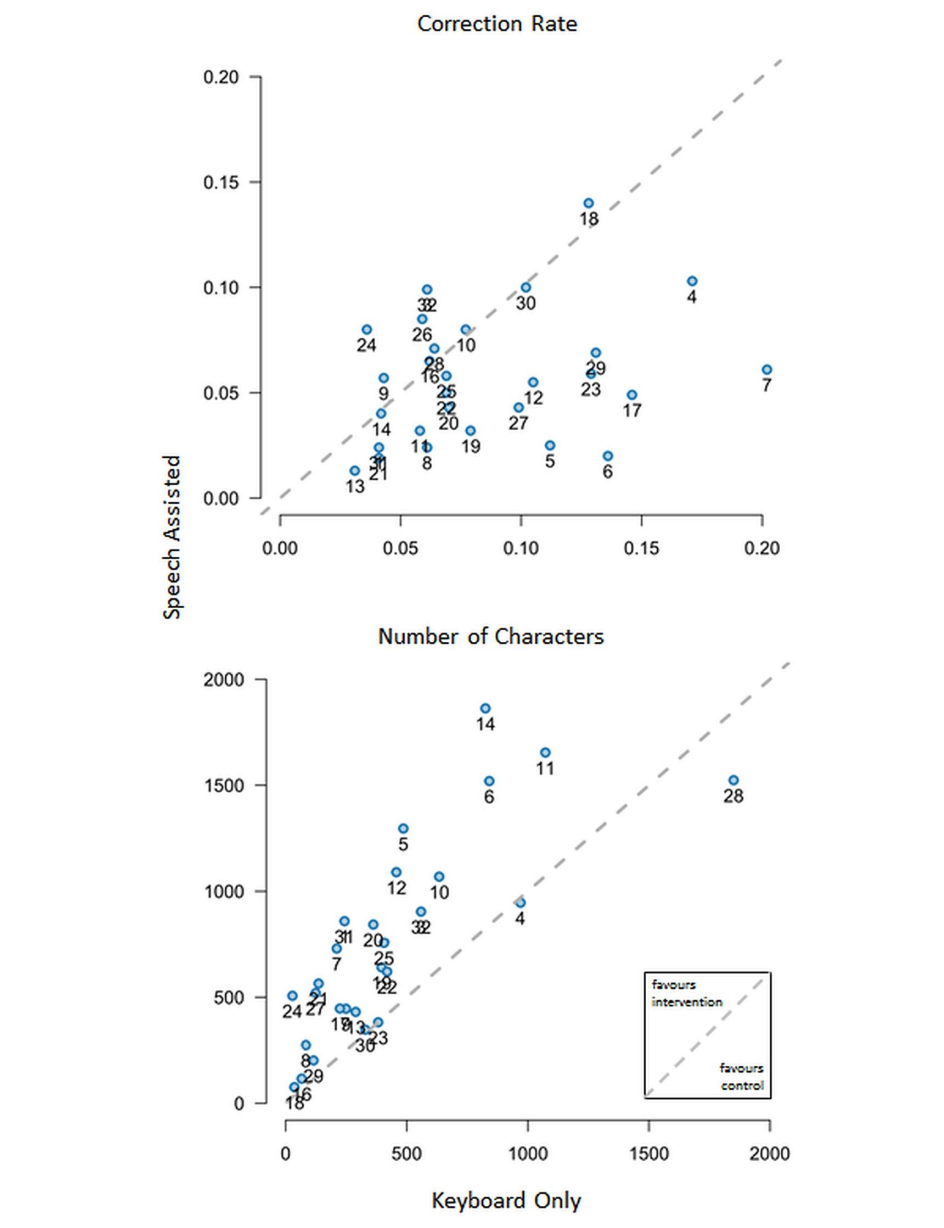
Per person analysis of correction rate and number of characters. Each dot represents one participant. The location of the dot indicates the sum of corrections per documented character (see text for further details) of control and intervention and the number of characters per report of control and intervention. A location above the dotted line indicates increased correction effort and increased number of characters per report when using the intervention.

While documenting a standardized text, 17 out of 28 (61%) participants were faster using speech. During productive use, 22 out of 28 participants (79%) were faster when using speech recognition (see Figure 5). The individual rate of corrections—sum of correction actions (delete, backspace, arrow keys, and mouse left click) per number of characters per report—was lower for speech-assisted documentation. The total number of characters per report was higher in the intervention group (speech recognition) (see Figure 6).

The measured total time of documentation was 37 hours and 18 minutes for the control group and 55 hours and 24 minutes for the intervention group. Using keyboard alone, 262,080 characters were entered into the study system compared to 465,785 characters during speech recognition availability. Tables 1 and 2 show an overview of captured data, including correction effort which is defined as the recorded keyboard strokes of the delete, backspace, and arrow keys, as well as mouse left clicks. Comparing control and intervention groups, the results show a significant increase in documentation speed, document length, and physician satisfaction. They also show a decreased correction rate and an increase in total documentation time secondary to the increased documentation amount.

## Discussion

### Principal Findings

Digital communication and electronic information exchange have become a part of everyday communication. Clinical documentation is a core aspect of the clinical profession and is more than a tool for efficient, maximized billing [9]. Nevertheless, in medicine the emphasis lies on paper-based documentation, being only assisted by electronic documentation. The reasons for this are the deficient quality and usability of electronic clinical documentation tools [1].

To enter information into the EHR, physicians rely on their ability to type or on the assistance of transcriptionists, but medical transcriptionists are a limited resource (eg, usually not available at the point of care or during nighttime). Therefore, the only alternatives for clinicians are pen-and-paper documentation, self-typing, or avoidance of documentation.

The objective of this study was to compare the impact of electronic speech recognition with self-typing based on measurement of documentation speed and volume, and user satisfaction. This explorative study based on 1455 captured medical documents demonstrated that the availability of Web-based medical speech recognition led to increased documentation speed, increased documentation amount, and higher physician satisfaction.

The study group consisted of native German speakers which favors good results in this German-based speech recognition system. Due to the correct grammar of a native speaker, word chains can be predicted by an ASR system thereby enhancing the recognition result. Foreign speakers face difficulties in using ASR systems mainly because of using incorrect grammar versus having an accent [10].

The study group was more satisfied when speech recognition was used in documentation. The reasons for increased satisfaction could be individual physiological factors like hand posture and typing speed. These factors greatly influence fatigability, finger pain, and various physiological aspects, which could explain different satisfaction levels [11]. On the other hand, due to the study group’s explorative composition, blocking and selection as well as stratification bias cannot be precluded.

### Requirements for Extensive Automatic Speech Recognition Usage in Clinical Routine

Electronic documentation tools are available on any desktop computer in hospitals. But the usual technical requirements for using speech recognition drastically reduce availability. The Web-based design of the study made speech recognition available on virtually any microphone-equipped desktop computer with a network connection. Documentation which is available everywhere in a hospital (also at the point of care) and simplified by suitable electronic tools leads to rapidly available individual texts. At the point of care, ASR systems have the greatest advantage over audio recordings since the documented text is electronically available day and night in real time [12].

Assuming availability issues were to be solved, remaining obstacles for widespread use of ASR in hospital settings include insufficient identification of employees with the new technology, slow learning curve, correction efforts, costs, and limited availability of microphones. Individual barriers and different usage types are reflected in the different gains when analyzing Web-based study ASRs on an individual level [2,13-15].

Improved human-machine interfaces, such as speech recognition or touch screen entry, could change the paradigm of paper-based documentation to full electronic documentation. This study addresses the question of whether typing alone or a combination of typing and speaking is a suitable human-machine interface in a general clinical setting. Autonomous clinical observations documented in real time are of utmost importance for the treatment process, and the acceptance of electronic documentation depends on the availability of these observations [16].

### Electronic Documentation

Despite obvious advantages, the change to complete electronic documentation is a matter of ongoing discussion; on one hand, electronic documentation promises increased efficiency and improvements in patient treatment through availability and readability. On the other hand, there are concerns of exchanging information electronically, such as the tracking of data to an individual, general data security, or compliance with local regulatory requirements [4]. An insufficient adaptation of systems for specific clinical requirements (eg, in pediatrics) immediately reduces the clinical usefulness, leading to avoidance and thereby manifesting the status quo [17].

In general, the use of electronic documentation in creating a clinical document is a multistage process that starts even before the patient has been seen by the physician: copying and pasting of personal information, importing of lab values, importing of the radiologist’s reports, or findings and reports of colleagues [18]. This can lead to fast but insufficiently individualized documentation, which can be troublesome. It is likely that certain clinical documentation tasks like informed consent will be exclusively documented electronically in the near future [19]. Electronic documentation may lead to a more complete documentation [20]. Consistent with this finding, the availability of a tool for the production of more complete documentation may be an explanation for the observed increase in documentation volume in our study.

### Strengths and Limitations

The strength of this study was being able to describe the effects of ASR availability on the clinical documentation process based on detailed data recorded by a computer program. This contrasts a significant number of studies on the topic which rely on perception-based data [1]. We present no proposal for a generally optimized clinical documentation process. The intervention affected only the self-typing by the medical personnel [2]. Provision via browser windows limited ASR availability issues to the presence of microphones. For study purposes, all computers involved in the study were equipped with a clip-on USB microphone.

Until recently, ASR training phases for new users were common. This was not done in this study system. Corrective user actions induce ASR system adaptation. Depending on the dictation style and the contents of the dictation, the errors made by ASR can be numerous. Therefore, different correction efforts and usage types could explain the scattering in Figure 6. This scattering was induced by the user, and the reaction of the system to the user. The reports created by the study participants were not evaluated for typos or misspellings. It was not the intention of the study to check the grammar quality of a medical report directly; this might be an objective for a follow-up trial. Another strength of the study was being able to measure the correction effort indirectly by counting correction-related user interactions. Effects resulting from insertion of text blocks were eliminated by limiting documentation speed to a physiologically sensible range.

As part of the personalization process, corrections have great impact on speech recognition systems. They modify statistical models and potentially add new words to the system. Corrective actions include deletion and insertion of words, replacement of system errors, inserting correct words through the keyboard, and modifying and changing text because of errors caused by voice. Personalization is the basis for good recognition results that are superior to the general recognition results of mobile devices [21]. The different usage patterns of using the mouse left-click button and the delete, backspace, and arrow keys were taken as an indicator of errors within the documentation. We emphasize that all necessary corrections, either using keyboard alone or ASR-assisted keyboard usage, were captured and included in the speed measurements. Maximum increased productivity can be reached if a trained system causing few errors is readily available [12,22].

Any study on the topic has to consider recruitment-induced biases. For this study, no staff groups or specializations were selected—recruitment was based on voluntary participation. A bias can arise easily owing to different specialties, different clinical knowledge, and different experiences with dictation in general [10]. Figure 5 illustrates the individual loss or gain when using the speech recognition system compared to self-typing. Overall, 6 individuals lost speed during the study period (productive use speed) while using the speech recognition system. Users 1 and 4 increased speed during productive use compared to standard text when using speech recognition, but due to high typing speed, there was no overall speed effect. User 28 was consistently slow compared to the rest of the group. For Users 18, 24, and 26, both typing speed and speech speed decreased. The underlying reasons for these observations were not explored. Although these 6 participants individually did not increase documentation speed, there was still an overall time-saving effect. Individual factors like slurred speech, dictation style, and dictation content may heavily influence the recognition result.

Together, all participants documented 727,865 characters (total documentation volume). Documenting this amount by the study group using the keyboard alone would have taken 104 hours. By adding speech to the documentation process, this time decreased to 87 hours. Therefore, the gain on the total documentation time would have been 17 hours. Despite this gain in documentation speed, the notable effect might not be decreased documentation time, but increased documentation volume.

Tools for efficient capturing of patient data and clinical observations are still underrepresented in clinical practice [23]. Even a trained typist will not achieve the same efficiency in capturing patient data on a mobile device’s touch screen as speech recognition or a digitizer pen could do day and night.

### Conclusions

We conclude that medical documentation with the assistance of Web-based speech recognition leads to an increase in documentation speed and amount, and enhances the participant’s mood when compared to self-typing. The remarkable effect might not be the time savings, but the increase in documentation volume. This study may be a starting point for further investigations where the overall efficiency of the documentation process, differences due to personal preferences, as well as aspects concerning quality of care and patient safety related to clinical documentation are explored.

The way medical documentation influences treatment quality needs to be understood better to choose the right mode of documentation and to help both the doctor and the patient. The continued exchange between health care personnel and technicians can facilitate a technological change in hospitals, and encourage technical advances leading to a more patient-centered treatment.

## Appendix

Multimedia Appendix 1 CONSORT-EHEALTH (v. 1.6.1) checklist.

## Abbreviations

ASR: automatic speech recognition
EHR: electronic health record
SSL: Secure Sockets Layer
WHO: World Health Organization
